# Supplementary material for: Molecular Characterization of Wilson’s Disease in Liver Transplant Patients: A Five-Year Single-Center Experience in Iran
Source: Diagnostics (Basel). 2025 Oct 1;15(19):2504. doi: 10.3390/diagnostics15192504 (PMC12523244; doi:10.3390/diagnostics15192504)
Supplement: Supplementary file 1 [file diagnostics-15-02504-s001.zip › diagnostics-3854749-supplementary.pdf]

Molecular Characterization of Wilson's Disease in Liver Transplant Patients: A Five-Year Single-Center Experience in Iran

Zahra Beyzaei [1], Melika Majed [1], Seyed Mohsen Dehghani [2], Mohammad Hadi Imanieh [2], Ali Khazaee [1], Bita Geramizadeh [1,3]*, Ralf Weiskirchen [4]*

Table S1. Features in human ATP7B protein and location of mutations.

Amino acid position	Feature	Amino acid substitution
23	PS	
61-124	HMA1	
67-69,72	Cu1	
		E127K
146-209	HMA2	
152-154,157	Cu2	
		R198G, Q228R
230-249	Dis	
		S254R
260-318	HMA3	
266-268,271	Cu3	M266L
322-355	Dis	
363-425	HMA4	
368-370,373	Cu4	
478	PS	
481	PS	
492-554	HMA5	
497-499,502	Cu5	
		L549V
567-630	HMA6	
573-575,578	Cu6	
		Q633P
652-1354	PtATPase	
654-675	TM1	
698-717	TM2	
725-745	TM3	V731E
		V750I, E754K
765-785	TM4	F771V
		P840L, V872E
920-942	TM5	
973-994	TM6	
983,985,1331	pCu	P992S; P992L; G998C
1027-1029,1097,1148-1150,1180,1220-1222,1242,1245,1248,1267,1270	pATP	E1082A
		E1293X, R1319X, I1321T
1323-1340	TM7	
		G1347V
1352-1371	TM8	

1398	PS	
		D1446Y

Abbreviations used: Dis, disordered; HMA, heavy-metal-associated domain; MB, metal-binding; pATP, putative ATP binding site; pCu, putative Cu binding site; PS, phosphorylation site (Serine; PtATPase, P-type_ATPase_Cu-like).

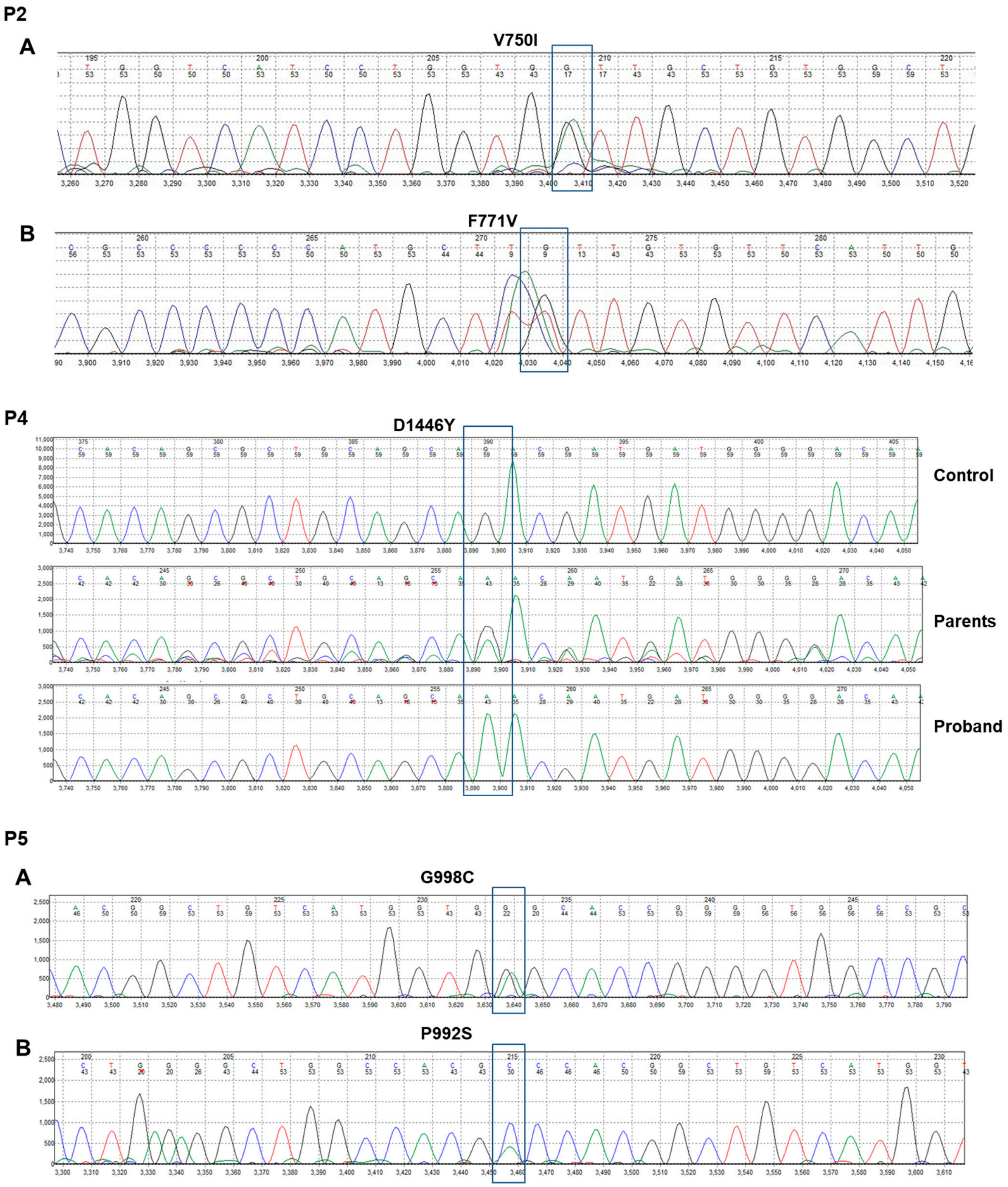

P16

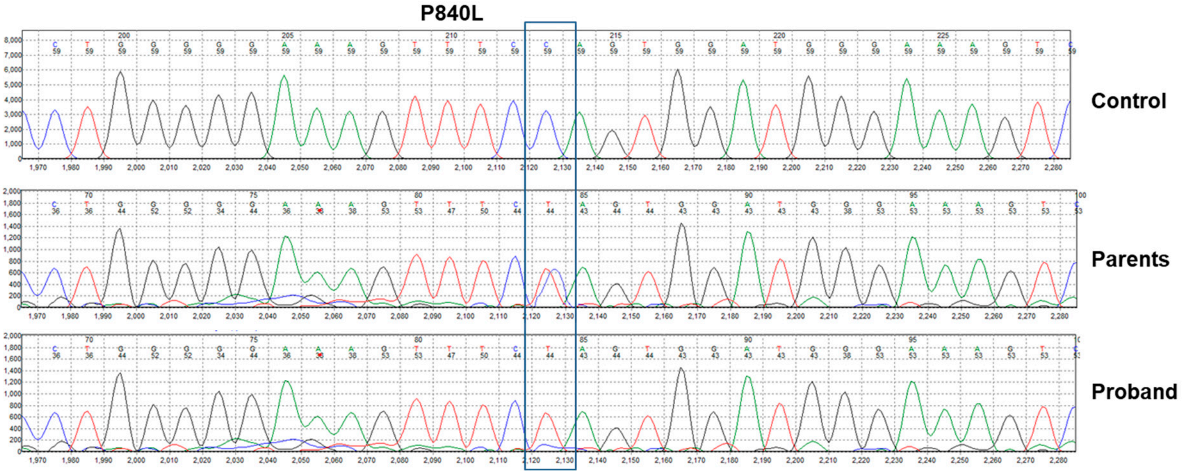
P840L
Control
Parents
Proband

P18

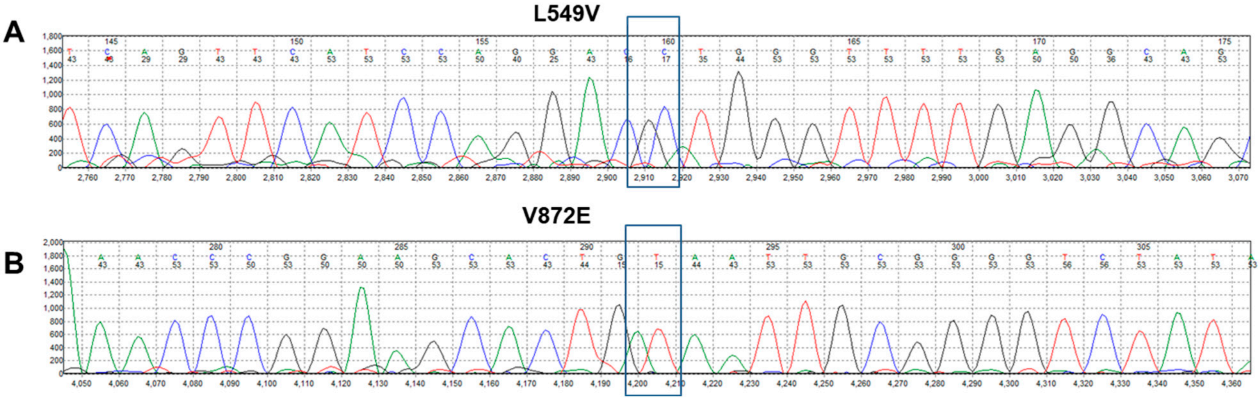
A
L549V
B
V872E

P24

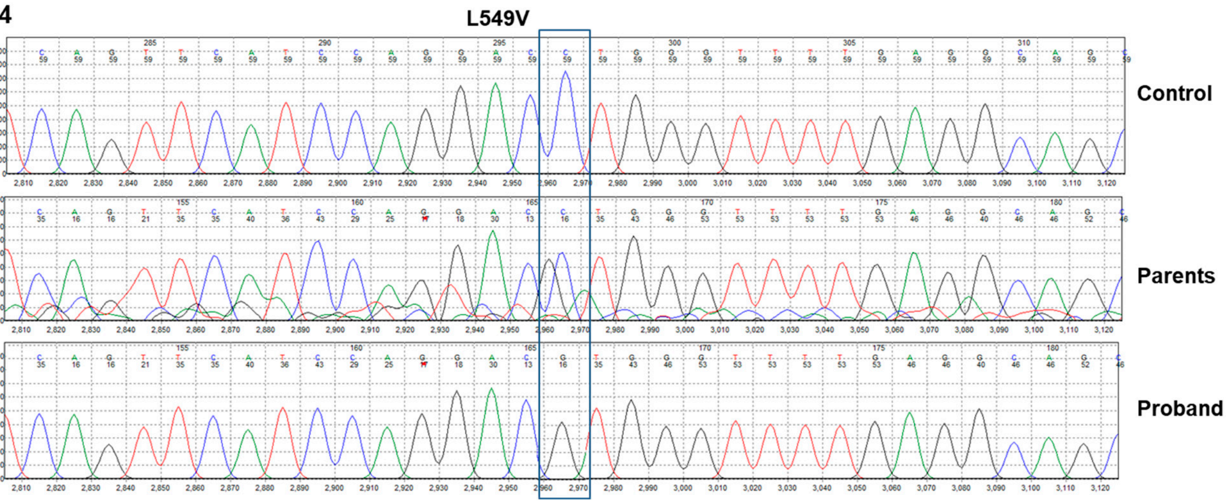
L549V
Control
Parents
Proband

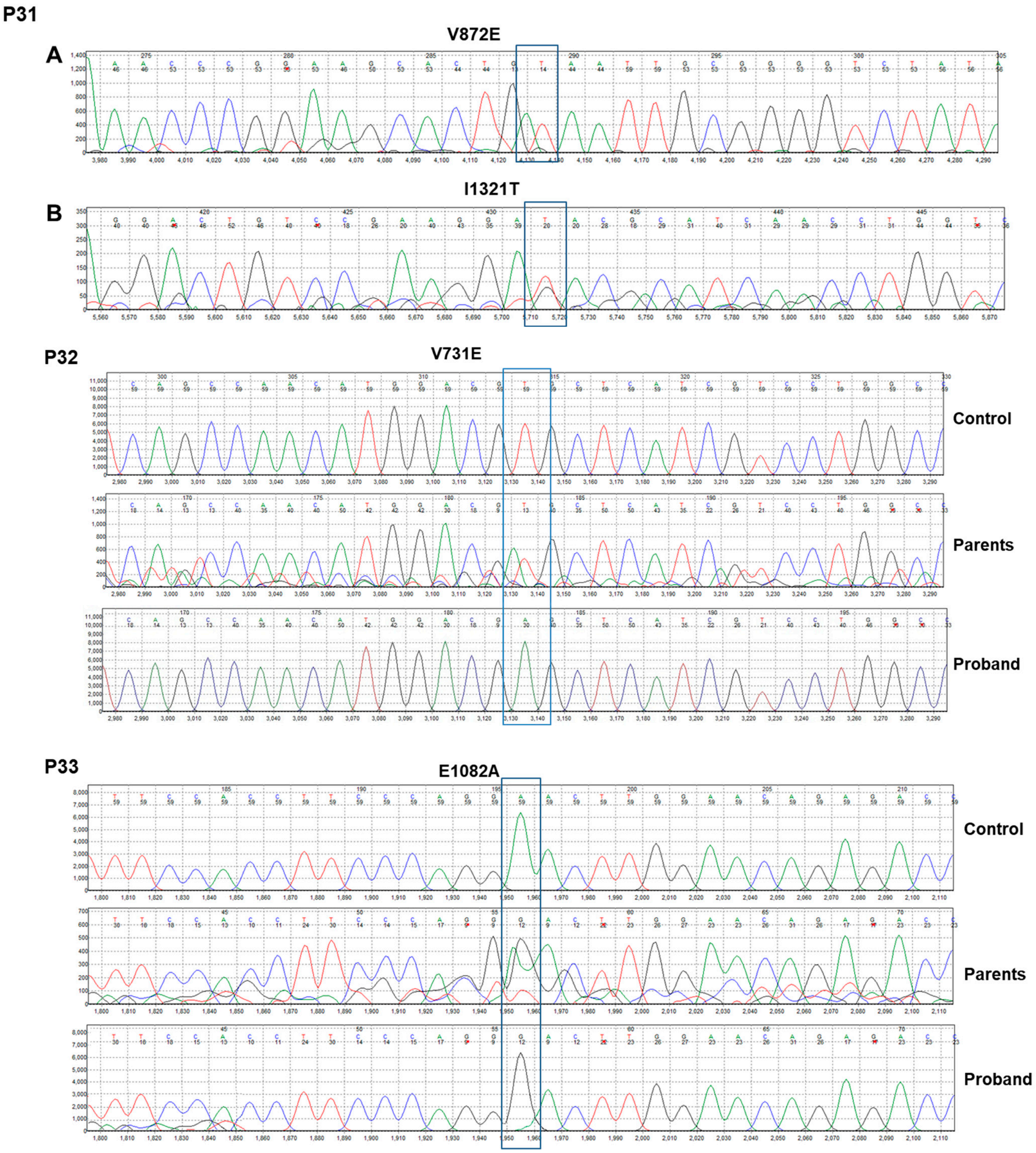
P31
V872E
A
I1321T
B
P32
V731E
Control
Parents
Proband
P33
E1082A
Control
Parents
Proband

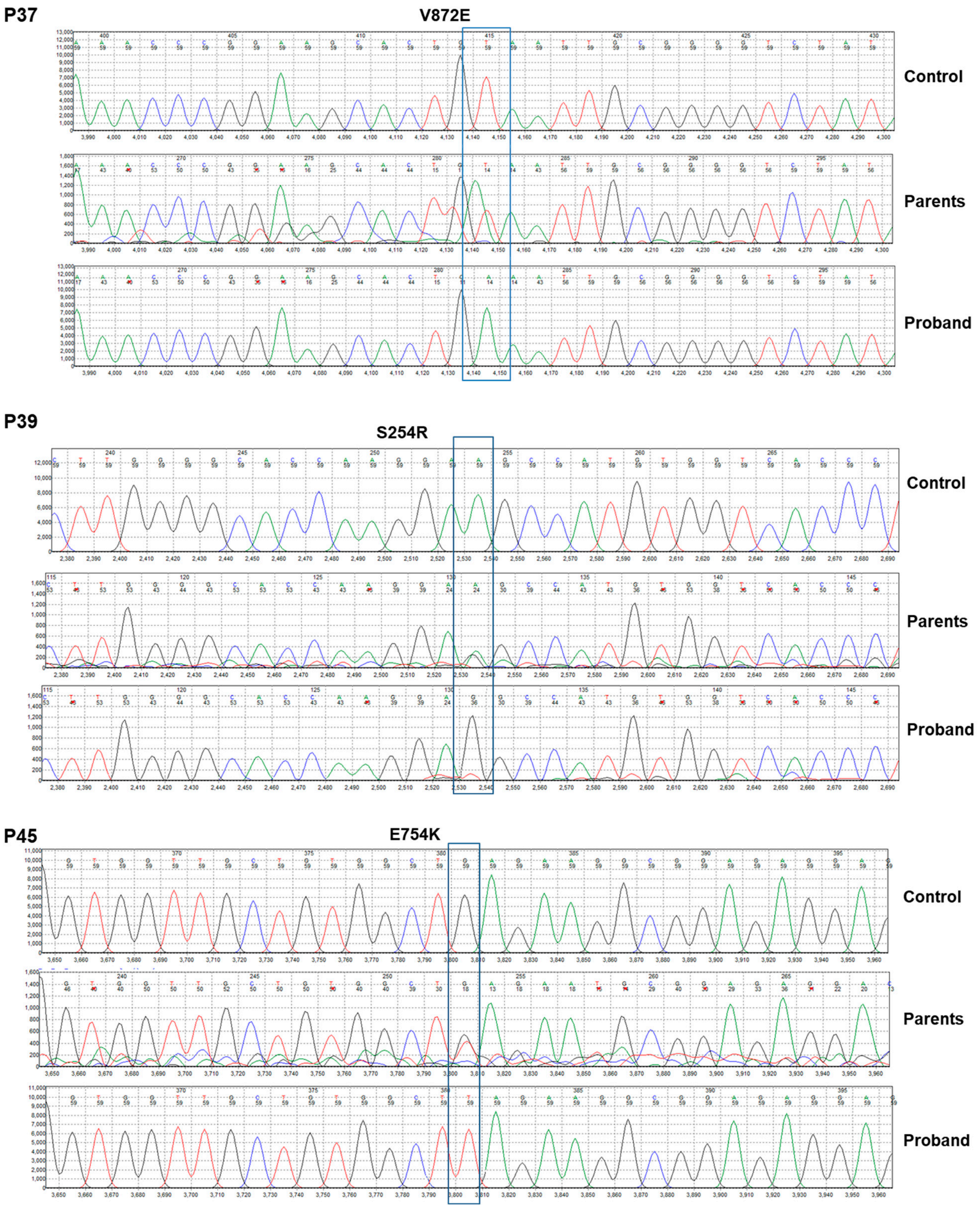

Figure S1. DNA sequence analysis of representative cases affected by homozygous or compound heterozygous *ATP7B* gene mutations. Sequencing results from patients P2 (V750I, F771V), P4 (D1446Y), P5 (G998C, P992S), P16 (P840L), P18 (L549V, V872E), P24 (L549), P31 (V872E, I1321T), P32 (V731E), P33 (E1082A), P37 (V872E), P39 (S254R), and P45 (E754K) are depicted. For patients carrying compound heterozygous mutations, the two variant sites are shown in **(A)** and **(B)**. The sequencing results from one control and one of the patients who carry the mutation in a heterozygous manner, as well as the result from the affected patients, are shown.

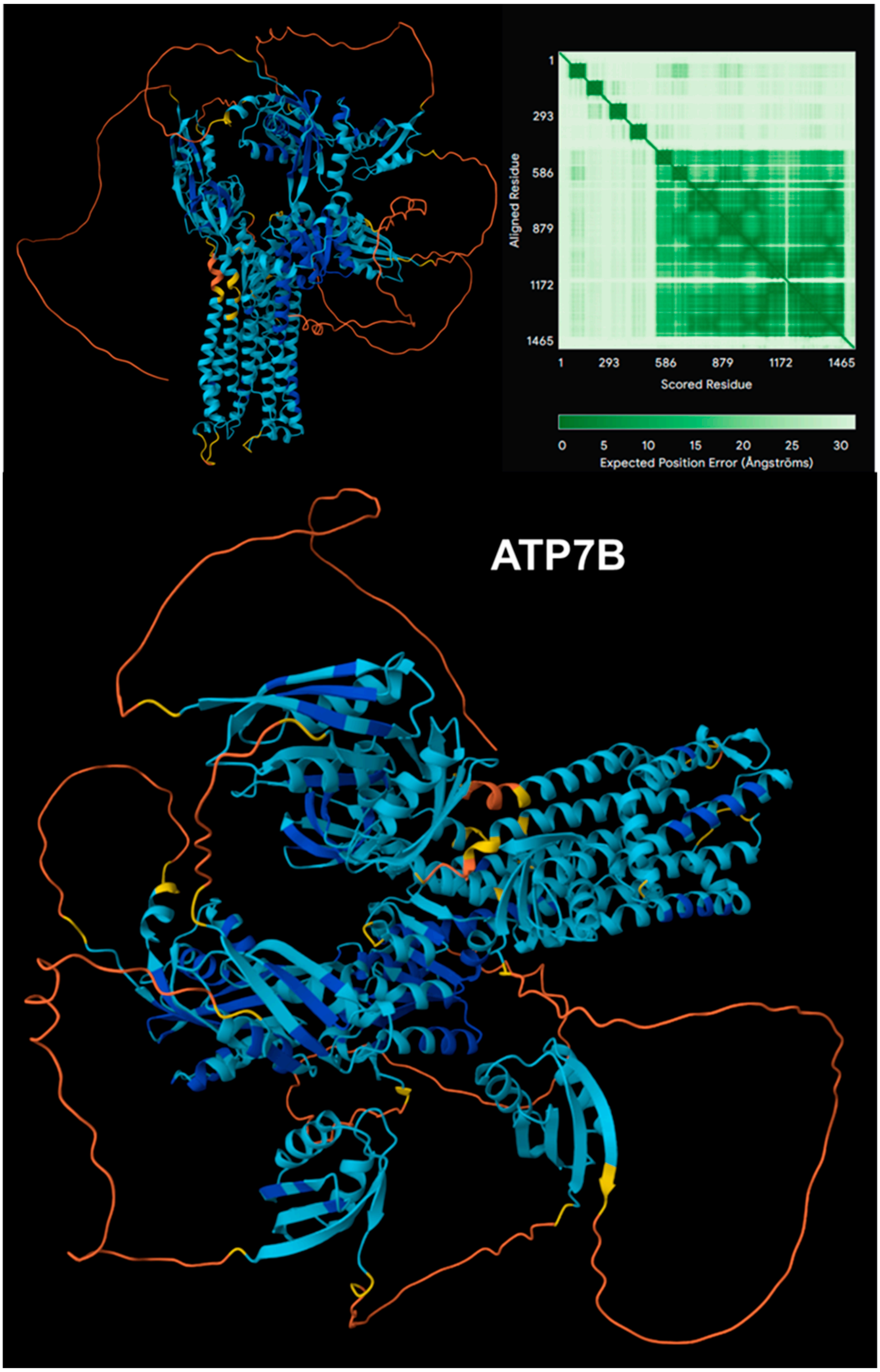

Figure S2. AlphaFold protein prediction for the human ATP7B protein. Shown is the output of AlphaFold prediction for the human ATP7B protein sequence. The predicted aligned error (PAE) indicates that the relative positions and orientations of most of the domains in the 3D structure are well defined.
